# Endometrial Cancer Patients: A Cohort Previous to Changes in Tumour Behaviour and Treatment Strategies

**DOI:** 10.5402/2011/950460

**Published:** 2011-12-18

**Authors:** F. K. L. Tournois, H. J. M. M. Mertens

**Affiliations:** Department of Obstetrics and Gynecology, Orbis Medical Centre, Postbus 5500, 6130MB Sittard, The Netherlands

## Abstract

Nowadays, the incidence of endometrial cancer is rising, especially of high-grade endometrial tumours. Recently, the FIGO classification of endometrial cancer has changed worldwide. Besides that, treatment strategies are changing. The purpose of this study was to analyse the adherence to the national guidelines of cancer treatment and to analyse patterns of disease relapse and survival. We focused on a group of patients (*n* = 191) with endometrial cancer, in a time period in which new treatment strategies are not yet completely implemented. Because of multiple upcoming changes in patient characteristics, tumour classification, as well as treatment regimens, a more heterogeneous cohort of patients diagnosed with endometrial cancer will appear. From now on, all those changes will have their effects on the followup of conventional endometrial cancer treatment. In our opinion, it is, therefore, valuable to have the current, more homogenous, cohort clearly described.

## 1. Introduction

Endometrial cancer is the most common cancer of the female genital tract in western countries [1]. Each year, approximately 17.8 per 100,000 women are diagnosed having endometrial cancer in the Netherlands [2]. Although endometrial cancer has the lowest mortality rates of all gynaecologic malignancies, still about 400 women yearly die from the disease in this country [3]. Nowadays, the incidence is rising due to an earlier diagnosis, a prolonged life expectancy, and an increasing incidence of risk factors such as nulliparity, obesity, and diabetes mellitus. Important prognostic factors are tumour stage, histopathology, histological grade, and depth of myometrial invasion [4, 5]. Recently, the FIGO stages of endometrial cancer have been revised worldwide [6, 7]. Besides that, treatment strategies are changing. The indications for adjuvant chemotherapy in cases of endometrial cancer are increasing and the mainstay of treatment is no longer (solely) total abdominal hysterectomy (TAH) and bilateral salpingo-oophorectomy (BSO) per laparotomy. Laparoscopic surgery has been proven to be safe and will become the treatment of choice in low-stage endometrial cancers [8–12].

The purpose of this study was to analyse the adherence to the (yet outdated) national protocols of cancer treatment and to analyse patterns of disease relapse and survival in patients having endometrial cancer in our hospital. We focused on a group of 191 patients with endometrial cancer in a time period during which new treatment strategies are not yet completely implemented.

## 2. Methods

A retrospective analysis was performed on all patients having endometrial cancer (*n* = 191), diagnosed between 2002 and 2010 in a Dutch teaching hospital in the south of the Netherlands. Patients and tumour characteristics were retrospectively obtained from hospital records. In the study period, patients with endometrial carcinoma were diagnosed according to the FIGO classification of 1988. Surgical and adjuvant treatment was performed according to the Dutch guidelines for endometrial carcinoma. We excluded patients with a sarcoma of the corpus uteri. The followup lasted till January 2011. According to national guidelines, the intention of curative treatment for endometrial cancer is a total abdominal hysterectomy with bilateral salpingo-oophorectomy (BSO). Adjuvant radiation- and/or chemotherapy were given based on the risk factors advised in the Dutch national guidelines of 2000. Radiation therapy was either vaginal brachytherapy alone or in combination with external beam radiation therapy (EBRT), depending on the FIGO stage [13, 14]. For the analysis of survival, we used the overall survival (OR) and disease-specific survival (DSS). In addition, the recurrence-free survival was calculated. Recurrence-free interval was defined as the time from surgical staging to the first evidence of recurrence (clinical, histological or radiological recurrence). The location of the recurrence was divided into three groups: (1) local recurrences of the vaginal vault, (2) regional recurrences in the distal vagina, pelvic cavity or pelvic lymph nodes, (3) distant metastasis. Survival analysis was performed using Kaplan-Meier curves. Differences between survival curves were assessed using the Mantel-Cox test. A *P*-value of < 0.05 was considered to be significant.

## 3. Results

A total of 191 patients were diagnosed having endometrial cancer. Thirteen patients (6.8%) were diagnosed in 2002, 20 (10.5%) in 2003, 35 (18.3%) in 2004, 30 (15.7%) in 2005, 17 (8.9%) in 2006, 18 (9.4%) in 2007, 34 (17.8%) in 2008, and 24 (12.6%) in 2009. The median age at initial diagnosis was 66.9 years (range 40–94 years). The mean time to followup was 37.6 months (range; 0–82 months; median 34.0 months). 

### 3.1. FIGO Stage

The majority of patients (*n* = 150; 78.5%) were diagnosed having FIGO stage I disease (IA in 33 (17.3%); IB in 78 (40.8%); IC in 39 (20.4%)). Ten patients (5.2%) were diagnosed having stage II disease (IIA in 3 (1.6%); IIB in 7 (3.7%)).

A total of 21 women (11.0%) were diagnosed having stage III disease (IIIA in 16 (8.4%); IIIB in 1 (0.5%); IIIC in 4 (2.1%)). Four patients (2.1%) were diagnosed having stage IV disease (all IVB).

Twenty patients (10.5%) died of endometrial carcinoma. Of all deaths, 3 (15.8%) were diagnosed having FIGO stage IB; 5 (26.3%) in IC, 5 (26.3%) in IIIA; 3 (15.8%) in IVB; 1 patient (5.3%) in the stages IIB, IIIB, and IIIC. Twenty-one patients (11.1%) died of causes other than endometrial carcinoma.

**Figure 1:**
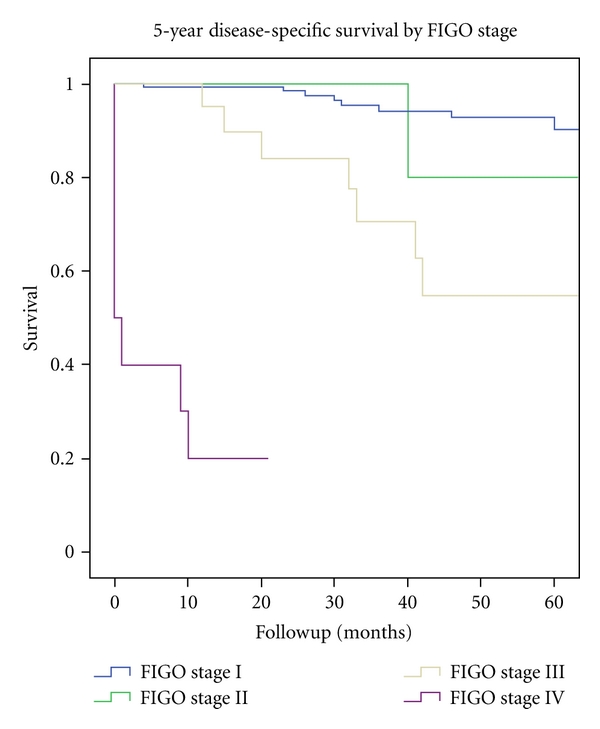
5-year disease-specific survival of *n* = 191 patients with endometrial cancer, divided by FIGO Stage (1988). Period of diagnosis 2002–2009.

The overall 5-year survival was 77.5% in FIGO stage I; 72.5% in FIGO stage II; 51.7% in FIGO stage III; 25% in FIGO stage IV disease (Table 1). The disease-free survival in FIGO stage I was 91.0%; in FIGO stage II 75.0%; in FIGO stage III 59.6%. The disease-specific survival was 92.6% in FIGO stage I and 80% in FIGO stage II disease (Figure 1). In FIGO stage III and IV, the disease-specific survival was equal to the overall survival. Overall and disease-specific survival in FIGO stage I was significantly better than in FIGO stage III and IV (*P* < 0.033).

### 3.2. Tumour Type and Grade

Adenocarcinoma of the endometrium was present in 165 patients (86.4%), papillary serous tumour in 18 (9.4%). Clear cell and mucinous tumours were present in, respectively, 6 (3.1%) and 2 (1.0%) patients. FIGO stage I disease was most seen in endometrioid adenocarcinomas (137 (86.2%) as opposed to 8 (44.4%) in papillary serous tumours).

Of all tumours, 69 (36.1%) were grade 1, 69 (36.1%) were grade 2, and 50 (26.2%) were grade 3 tumours. In FIGO stage III, the percentage of grade 3 tumours was higher than in FIGO stage I disease (grade I, II, and III in FIGO stage I was 41.3%, 38.0%, and 20.7% as opposed to 14.3%, 33.3%, and 52.4% in FIGO stage III). In stage IV, all tumours were high grade.

**Figure 2:**
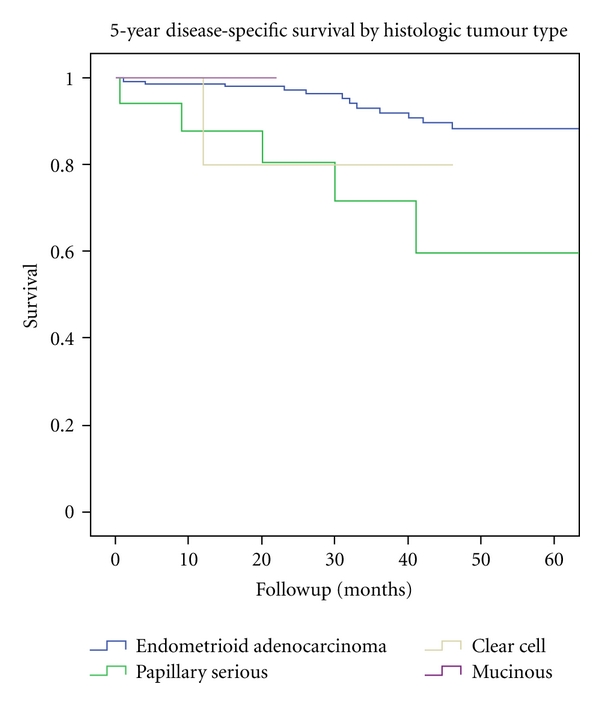
5-year disease-specific survival of *n* = 191 patients with endometrial cancer, divided by histologic tumour type. Period of diagnosis 2002–2009.

The overall 5-year survival was 73.5% in endometrioid adenocarcinomas and 59.6% in papillary serous tumours. The disease-specific survival in endometrioid tumours was 87.7% and in serous tumours 59.6% (Figure 2). The disease-free survival was 89.2% and 68.6%, respectively. Both disease-specific and disease-free survival curves were significantly better in endometrioid tumours (*P* = 0.002 and *P* = 0.02, resp.).

**Figure 3:**
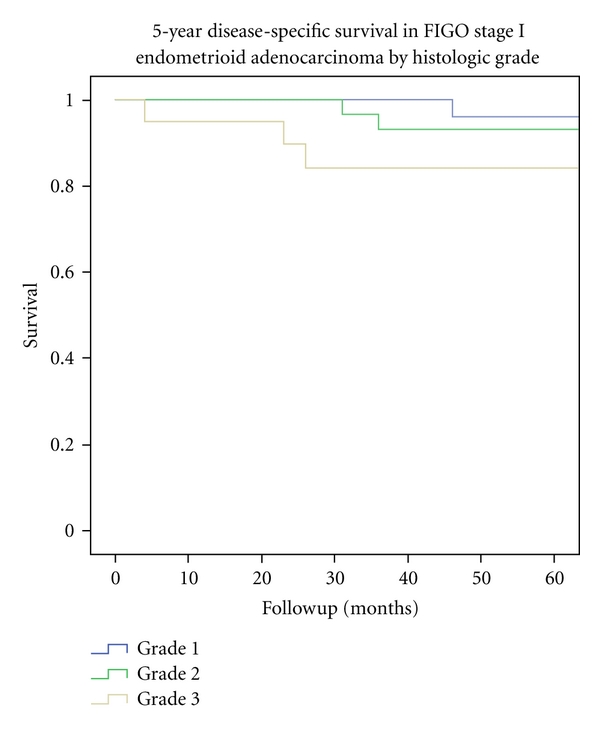
5-year disease-specific survival within FIGO stage I endometrioid adenocarcinoma *n* = 136, divided by histologic tumour grade. Period of diagnosis 2002–2009.

In FIGO stage I adenocarcinomas, the overall 5-year survival in grade 1 tumours was 90.7%, 74.5% in grade 2, and in 59.8% grade 3 tumours. Disease-specific survival was 96.0% in grade 1 tumours, 93.1% in grade 2 tumours, and 84.1% in grade 3 tumours (Figure 3). Disease-free survival was 92.4% in grade 1 tumours, 90.7% in grade 2, and 88.2% in grade 3 tumours. Overall- and disease-specific survival was significantly better in grade 1 tumours versus grade 3 tumours (*P* = 0.009 and *P* = 0.11, resp.).

### 3.3. Therapy

Of all patients, 183 (95.8%) underwent a surgical procedure. Eight patients (4.1%) did not get any form of surgical therapy. In all records, the reason for noninvasive management was described (2 patients died before therapy could have been started; 5 refused therapy because of severe comorbidity (2 of those 5 received hormonal therapy); 1 refused therapy on personal arguments).

In 178 women (97.3%), the surgical procedure was according to national guidelines. Of all patients that were not treated according to the guidelines, 1 patient underwent a BSO without hysterectomy because of massive extrauterine extension of the tumour into the distal vagina and 4 patients underwent a hysterectomy without the adnexa (because of multiple adnexal adhesions (*n* = 2) or a vaginal procedure (*n* = 2)). In all cases, the reason for not adhering to national guidelines was documented.

### 3.4. Lymphadenectomy

During the study period, we did not routinely perform a lymphadenectomy in endometrial cancer patients unless there were palpable lymph nodes. Three (1.7%) patients underwent a lymph node extirpation. All of them had serous tumours. The number of lymph nodes that were dissected was 1–5 nodes. All nodes were negative. There was no additional perioperative morbidity.

From late 2009 onwards, we performed a complete lymph node dissection in two patients with high-grade endometrial cancer. One of them had a clear cell endometrial carcinoma, the other a papillary serous endometrial carcinoma. In these cases, a gynaecologic oncologist performed the procedure (dissected lymph nodes 20–29).

Only the lymph nodes of the patient with the clear cell tumour were positive (4/29). She was upgraded from FIGO stage IC to FIGO stage IIIC and received adjuvant radiotherapy and chemotherapy.

### 3.5. Adjuvant Therapy

In 9 cases (4.9%), adjuvant radiotherapy was not given according to the Dutch national guidelines of 2000. Three patients were not treated with adjuvant radiotherapy because of severe comorbidity. One patient did not wish to receive adjuvant therapy due to personal reasons. Two patients died before start of adjuvant therapy. For 3 patients, there was no documented argumentation.

### 3.6. Recurrence

Of all recurrences (*n* = 22 (11.5%)), there were local recurrences in 7 patients (31.8%), regional recurrences in 2 (9.1%), and newly discovered distant metastases in 13 patients (59.1%). Most common areas for metastatic disease were lymph nodes, lungs, abdomen, and skeleton.

In FIGO stage I disease, local recurrences were seen in 5 patients (41.7%), regional recurrences in 1 patient (8.3%), and distant metastases in 6 patients (50.0%). In FIGO stage II, 1 patient (50.0%) had a local recurrence and 1 (50.0%) had a distant metastasis. In FIGO stage III, 6 patients (75.0%) had distant metastases and 1 patient (12.5%) developed a local recurrence.

In FIGO stage I with adjuvant radiotherapy (*n* = 40), 1 patient (2.5%) developed a regional recurrence and 4 patients (10.0%) developed metastatic disease. In FIGO stage II with adjuvant radiotherapy (*n* = 10), 1 patient (10.0%) developed a local recurrence, 1 (10.0%) a regional recurrence, and 1 (10.0%) metastatic disease. In FIGO stage III with adjuvant radiotherapy (*n* = 20), 1 patient (5.0%) developed a local recurrence, and 5 patients (25.0%) distant metastases. Nine patients (7.1%) had recurrent disease in the group that did not receive adjuvant radiotherapy (*n* = 126).

## 4. Discussion

A description of a cohort of patients having endometrial cancer is not unique. The patient characteristics, tumour characteristics, survival, and recurrence outcomes in this study are in accordance with large (multicenter) international studies [15–17]. Still, we consider the results of this study very important, because the treatment of patients having endometrial cancer is changing. This local study will be the last before new surgical and adjuvant therapy treatment strategies are fully implemented and the FIGO stages have been reclassified.

A Dutch study in the same region has shown that the incidence of endometrial cancer is increasing [18]. Besides that, the relative incidences of high-grade tumours and serous tumours are increasing as well. The increasing incidence of overweight patients and obesity in western countries contributes to a higher cancer rate in general and endometrial cancer in particular. There are many other possible explanations why the incidence is rising, especially in the high-grade endometrial tumours; for instance, higher life expectancy, declining fertility rates, and better availability of valid diagnostic procedures [19, 20]. The characteristics of patients diagnosed and treated for endometrial cancer will definitely change within the next few years.

Besides the changes in patients' and tumour characteristics, surgical treatment strategies have also undergone some important changes. Since 2010, in low-grade endometrial cancer, a laparoscopic approach is preferred to a laparotomic surgical procedure. Also, a lymphadenectomy is believed to be beneficial in a “selective group” of patients with endometrial cancer [21–24]. Although there is still discussion about the specific group of patients, the extent of the procedure and its benefit, it is clear that surgical treatment strategies will be different from now on. In our region, from 2010 onwards, a lymphadenectomy is routinely recommended in high-grade endometrioid, papillary serous, and clear cell tumours, unless it is contraindicated due to severe comorbidity. In all these cases, a gynaecologic oncologist has to perform this procedure.

Aside from these changes in surgical treatment strategies, the indications for adjuvant therapy following surgery are also expanding. Particularly, there is a lot of ongoing research to determine the value of and the indication for chemotherapy or chemoradiation therapy in women with high-risk endometrial cancer [13, 25–35].

In the very last period of inclusion for this study, we were already routinely performing a complete lymphadenectomy. The FIGO stage of this patient was upgraded and chemotherapy was added to the adjuvant therapy. In the future, these changes in adjuvant treatment will have influences on the patients' morbidity.

Boll and coauthors have found that comorbidity decreases the likelihood of receiving adjuvant radiotherapy in patients with FIGO stage I endometrial cancer that were qualified to receive adjuvant radiotherapy according to the Dutch national guidelines [36]. It is conceivable that the previously described changes in patients' characteristics, together with more invasive surgical treatments with routine lymphadenectomy, the possibility of a laparoscopic procedure and a decreased likelihood of receiving adjuvant therapy because of severe comorbidity will make the group of patients with endometrial cancer very heterogeneous. Taking this into consideration, the adherence to (national) guidelines may become less. Therefore, future studies like this will become difficult because of the heterogeneity of the group of patients treated for endometrial cancer.

Yet from today, all those changes will have their effects on the followup of endometrial cancer treatment. In our opinion, it is, therefore, valuable to have the current, more homogenous, cohort clearly described.

## Figures and Tables

**Table 1:** 5-year survival rates of *n* = 191 patients with endometrial carcinoma diagnosed between 2002 and 2009. Subdivided into histological tumour type, FIGO stage and histological tumour grade.

	OS^a^	DSS^b^	DFS^c^
Histology			
Endometrioid	73.5%	87.7%	89.2%
Papillary serous	59.6%	59.6%	68.6%
Clear cell^d^	40.0%	80.0%	60.0%

FIGO			
Stage I	77.5%	92.6%	91.0%
Stage II	72.5%	80.0%	75.0%
Stage III	51.7%	51.7%	59.6%
Stage IV	25.0%	25.0%	—

Tumour grade (within FIGO stage I endometrioid)			
Grade 1	90.7%	96.0%	92.4%
Grade 2	74.5%	93.1%	90.7%
Grade 3	59.8%	84.1%	88.2%

^a^: 5-year overall survival.

^b^: 5-year disease-specific survival.

^c^: 5-year disease-free survival.

^d^: maximum follow-up: 46 months.
